# N-Succinylaspartic-Acid-Conjugated Riluzole Is a Safe and Potent Colon-Targeted Prodrug of Riluzole against DNBS-Induced Rat Colitis

**DOI:** 10.3390/pharmaceutics15112638

**Published:** 2023-11-16

**Authors:** Jaejeong Kim, Changyu Kang, Jin-Wook Yoo, In-Soo Yoon, Yunjin Jung

**Affiliations:** College of Pharmacy, Pusan National University, Busan 46241, Republic of Korea; wowjd9669@naver.com (J.K.); whale10000@naver.com (C.K.); jinwook@pusan.ac.kr (J.-W.Y.); insoo.yoon@pusan.ac.kr (I.-S.Y.)

**Keywords:** riluzole, colon-targeted drug delivery, colitis, prodrug, N-succinylated acidic amino acids

## Abstract

In our previous study, riluzole azo-linked to salicylic acid (RAS) was prepared as a colon-targeted prodrug of riluzole (RLZ) to facilitate the repositioning of RLZ as an anticolitic drug. RAS is more effective against rat colitis than RLZ and sulfasalazine, currently used as an anti-inflammatory bowel disease drug. The aim of this study is to further improve colon specificity, anticolitic potency, and safety of RAS. N-succinylaspart-1-ylRLZ (SAR) and N-succinylglutam-1-ylRLZ (SGR) were synthesized and evaluated as a “me-better” colon-targeted prodrug of RLZ against rat colitis. SAR but not SGR was converted to RLZ in the cecal contents, whereas both conjugates remained intact in the small intestine. When comparing the colon specificity of SAR with that of RAS, the distribution coefficient and cell permeability of SAR were lower than those of RAS. In parallel, oral SAR delivered a greater amount of RLZ to the cecum of rats than oral RAS. In a DNBS-induced rat model of colitis, oral SAR mitigated colonic damage and inflammation and was more potent than oral RAS. Moreover, upon oral administration, SAR had a greater ability to limit the systemic absorption of RLZ than RAS, indicating a reduced risk of systemic side effects of SAR. Taken together, SAR may be a “me-better” colon-targeted prodrug of RLZ to improve the safety and anticolitic potency of RAS, an azo-type colon-targeted prodrug of RLZ.

## 1. Introduction

Inflammatory bowel diseases (IBD) such as ulcerative colitis (UC) and Crohn’s disease (CD) are chronic and intractable inflammatory disorders of the gastrointestinal (GI) tract [1]. Unlike UC, which occurs mainly in the distal part of the large intestine and whose pathological state is limited to the innermost mucosal layer, the pathological lesions of CD develop throughout the GI tract and affect the deeper intestinal wall up to the muscular layer [1,2].

Drug therapies for IBD focus on the remission of inflammation and maintenance [2,3]. Current anti-IBD drugs are classified as small-molecular organic medicines, aminosalicylates, and glucocorticoids; immunosuppressants including c-Jun N-terminal kinase inhibitors; and biopharmaceuticals (biologics) including anti-tumor necrosis factor, anti-α_4_β_7_ integrin, and anti-interleukin (IL)-12 and IL-23 agents [2,4,5]. In IBD pharmacotherapy, biologics elicit better therapeutic performance than small-molecule drugs with low anti-IBD efficacy and limitations in long-term use owing to serious side effects [5]. The market share of biologics in the drug market for IBD treatment is increasing. However, a considerable proportion of patients with IBD are not satisfactorily responsive to biologics, and their side effects are not negligible, along with high medical costs and poor patient compliance due to parenteral use [6,7,8]. Therefore, although biologics provide a suitable therapeutic option for IBD, there is still a substantial unmet medical need for small-molecule anti-IBD drugs with improved efficacy and safety.

Colon-targeted delivery of a drug, in which the drug is delivered specifically to the large intestine, without systemic absorption and metabolic loss during transit of the stomach and small intestine upon oral administration, is frequently employed to enhance the therapeutic activity of anti-IBD drugs [9]. In general, this technique tends to increase therapeutic availability at the target site and decrease the systemic absorption of a drug compared to conventional oral drug delivery [10]. Therefore, in addition to its therapeutic advantages, colon-targeted drug delivery (CTDD) is a feasible strategy to circumvent safety issues caused by the systemic side effects of anti-IBD drugs [11,12]. Based on these benefits, CTDD is utilized to facilitate the repositioning of a drug as an anti-IBD drug [13].

In our previous study [13], to reposition riluzole (RLZ), an FDA-approved anti-amyotrophic lateral sclerosis drug, as an anti-IBD drug, RLZ azo-linked to salicylic acid (RAS) was synthesized as a colon-targeted prodrug and evaluated as an anti-IBD drug in a DNBS-induced rat colitis model. Compared with the oral administration of RLZ, oral RAS was therapeutically more potent against rat colitis and significantly reduced the systemic absorption of RLZ. To further improve RAS’s anticolitic potency and safety, a more hydrophilic colon-targeted prodrug of RLZ was designed for greater colonic delivery efficiency and restriction of RLZ systemic absorption. For this purpose, instead of using SA as a colon-specific carrier (for RAS), the aromatic amine group in RLZ was coupled with a carboxylic acid group in acidic amino acids, such as aspartic acid (Asp) and glutamic acid (Glu), to form an amide bond, followed by the N-succinylation of aliphatic amines in acidic amino acids [14]. N-Succinylation was employed to further increase hydrophilicity and biochemical stability. The simple conjugates produced through the coupling of a drug with Asp or Glu tends to be metabolized (deconjugated) to the parent drug by an enzyme (such as an aminopeptidase) in the small intestine, thus prematurely releasing the drug before reaching the target site in the large intestine [14]. Succinic acid is an endogenous and safe molecule, similar to amino acids. In this study, hydrophilic RLZ derivatives were synthesized and evaluated for colon specificity and anticolitis activity in a DNBS-induced rat colitis model and compared to RAS.

## 2. Materials and Methods

### 2.1. Materials

Sulfasalazine (SSZ) and succinic anhydride were purchased from the Tokyo Chemical Industry (Tokyo, Japan). RLZ, 1,1′-carbonyldiimidazole (CDI), 4-benzyl N-(t-Boc)-aspartic acid, and 5-benzyl N-(t-Boc)-glutamic acid were purchased from Ambeed, Inc. (Arlington Heights, IL, USA). Solvents for synthetic reaction and high-performance liquid chromatography (HPLC) analysis were provided by Junsei Chemical Co. (Tokyo, Japan) and DAEJUNG Chemicals & Metals Co., Ltd. (Gyeonggi, Republic of Korea), respectively. Phosphate buffer saline (pH 7.4, PBS) was purchased from Thermo Fisher Scientific (Waltham, MA, USA). All other chemicals were commercially available, reagent-grade products. Enzyme-linked immunosorbent assay (ELISA) kits for the quantification of cytokine-induced neutrophil chemoattractant-3 (CINC-3) and interleukine-10 (IL-10) were obtained from R&D Systems (Minneapolis, MN, USA).

Thin-layer chromatography (TLC) plates (silica gel F254s, Merck Millipore, Burlington, MA, USA) were used to analyze synthesis of RLZ derivatives, and the spot on TLC plates was detected using an ultraviolet lamp (254 nm). A Varian Fourier transform infrared (FT-IR) spectrophotometer (Palo Alto, CA, USA) and Varian AS 500 nuclear magnetic resonance (NMR) spectrometer were used to record the IR and 1H-NMR spectra, respectively. Tetramethylsilane was used as an internal reference compound for presenting the chemical shift (ppm) in the 1H-NMR spectra. Electrospray ionization mass spectrometry (ESI-MS, Agilent 65360 Q-TOF instrument, Santa Clara, CA, USA) was conducted to analyze molecular weights of products.

### 2.2. Synthesis of (N-succinyl-L-aspart-1-yl)riluzole and (N-succinyl-L-glutam-1-yl)riluzole

The 4-Benzyl N-(t-Boc)-aspartic acid (323.3 mg, 1 mmol) was dissolved in 15 mL of acetonitrile (ACN), followed by the addition of CDI (202.6 mg, 1.25 mmol), which was stirred at 25 °C for 2 h, and then RLZ (140.4 mg, 0.6 mmol) and triethylamine (TEA, 0.3 mL) were added to the reaction mixture. After 24 h, the solvent was removed through evaporation. The residue was dissolved in ethyl acetate (EA), washed three times with 0.1 M HCl and 5% Na_2_CO_3_ solution, and dried over anhydrous Na_2_SO_4_. After removal of EA, trifluoroacetic acid (TFA)/dichloromethane (DCM) (4.5/5.5, 30 mL) was added to the residue for reaction at 25 °C for 45 min, followed by removal of TFA/DCM and subsequent addition of 1 M NaOH (10 mL) at 40 °C for 2 h. The alkaline solution was washed with EA and acidified to obtain the product L-aspart-1-ylRLZ (2, AR) as a white precipitate. AR (M.W.: 349.28); yield: 65%; melting point: 163 °C; IR (nujol mull): ν_max_ (cm^−1^) = 1689 cm^−1^ (-C=O, carboxylic), 1612 cm^−1^ (-C=O, amide); ^1^H-NMR (DMSO-d_6_): 8.17 (s, 1H), 7.92 (d, *J* = 8.8 Hz, 1H), 7.45 (dd, *J* = 8.8, 1.7 Hz, 1H), 4.11 (m, 1H), 2.81 (dd, *J* = 16.6, 4.3 Hz, 1H), and 2.67 (dd, 1H). ESI-MS [M-H]-:*m*/*z* = 348.01. To synthesize N-succinylaspart-1-ylRLZ (SAR), succinic anhydride (300 mg, 3 mmol) was added to a solution of 1 (ACN, 15 mL) in the presence of TEA (0.3 mL), stirring at 55 °C for 4 h. After removal of the solvent, the residue was treated with 1 M NaOH (10 mL) at 40 °C for 2 h. The alkaline solution was acidified to obtain the final product of N-succinylaspart-1-ylRLZ (4, SAR) as a white precipitate. SAR (M.W.: 449.35); yield: 74%; melting point: 202 °C (decomp); IR (nujol mull): ν_max_ (cm^−1^) = 1699 cm^−1^ and 1657 cm^−1^ (-C=O, carboxylic), 1611 cm^−1^ and 1557 cm^−1^ (-C=O, amide); ^1^H-NMR (DMSO-d_6_): ^1^H-NMR (DMSO-d_6_): 8.13 (d, *J* = 1.7 Hz, 1H), 7.84 (d, *J* = 8.8 Hz, 1H), 7.43 (dd, *J* = 8.7, 1.8 Hz, 1H), 4.83 (m, 1H), 2.79 (dd, *J* = 16.8, 6.1 Hz, 1H), 2.65 (dd, *J* = 16.7, 7.5 Hz, 1H), 2.50 (t, *J* = 1.7 Hz, 2H), 2.43 (m, 2H). ESI-MS [M-H]-:*m*/*z* = 448.07.

**Figure 1:**
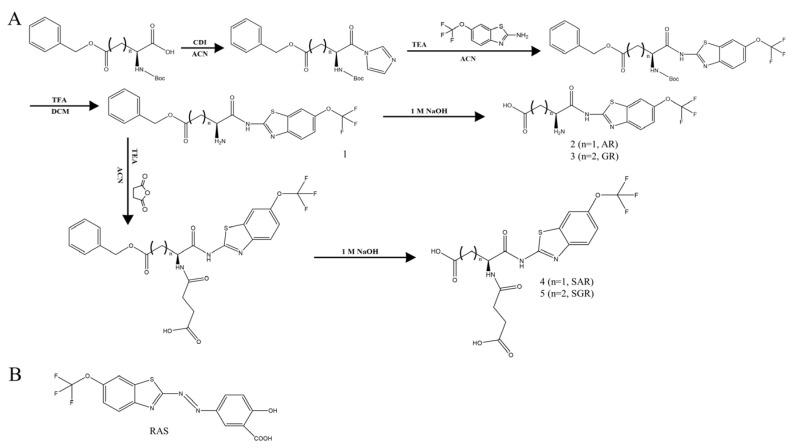
Synthesis of SAR and SGR and structure of RAS. (**A**) Synthetic scheme of SAR and SGR. SAR: (N-succinyl-L-aspart-1-yl)riluzole, SGR: (N-succinyl-L-glutam-1-yl)riluzole, AR: N-(L-aspart-1-yl)riluzole, GR: N-(L-glutam-1-yl)riluzole, TEA: triethylamine, CDI: 1,1′-carbonyldiimidazole, ACN: acetonitrile, and DCM: dichloromethane. (**B**) Structure of RLZ azo-linked salicylic acid (RAS).

L-Glutam-1-ylRLZ (3, GR) was synthesized from 5-benzyl N-(t-Boc)-glutamic acid using the same synthetic method as AR. GR (M.W.: 363.31); yield: 69%; melting point: 169 °C; IR (nujol mull): ν_max_ (cm^−1^) = 1673 cm^−1^ (-C=O, carboxylic), 1613 cm^−1^ (-C=O, amide); ^1^H-NMR (DMSO-d_6_): 8.18 (s, 1H), 7.88 (d, *J* = 8.8 Hz, 1H), 7.47 (dd, *J* = 8.7, 1.9 Hz, 1H), 2.42 (m, 2H), 2.12 (m, 2H). ESI-MS [M-H]-:m/z = 362.03. N-Succinylglutam-1-ylRLZ (5, SGR) was synthesized using the same method as that used for SAR. SGR (M.W.: 463.38); yield: 76%; melting point: 258 °C (decomp); IR (nujol mull): ν_max_ (cm^−1^) = 1689 cm^−1^ (-C=O, carboxylic), 1616 cm^−1^ and 1584 cm^−1^ (-C=O, amide); ^1^H-NMR (DMSO-d_6_): 8.27 (d, *J* = 7.8 Hz, 1H), 8.11 (d, *J* = 1.7 Hz, 1H), 7.81 (d, *J* = 8.8 Hz, 1H), 7.41 (dd, *J* = 8.8, 2.4 Hz, 1H), 4.22 (m, 1H), 2.61 (t, *J* = 7.6 Hz, 2H), 2.43 (m, 2H), 2.41 (m, 2H), 2.11(m, 1H), 1.89 (m, 1H). ESI-MS [M-*H*]^−^:*m*/*z* = 462.05. Synthetic scheme of AR, GR, SAR, and SGR is shown in Figure 1A. RLZ azo-linked to salicylic acid (RAS) was synthesized as described previously [13].

### 2.3. HPLC Analysis

The HPLC system consisting of a Gilson Model 306 pump, 151 variable UV detectors, and 234 auto-injector (Gilson, Middleton, WI, USA) was used to analyze the drugs in the samples. Chromatographic separation was performed using A C18 column (Hector, Theale, Berkshire, UK; 250 × 4.6 mm, 5 μm). Before applying samples for HPLC analysis, the samples obtained from each experiment were diluted appropriately, and then were subjected to filtration through membrane filters (0.45 μm). The HPLC analysis was performed with mobile phases A and B consisting of distilled water and acetonitrile at 4:6, *v*/*v* and 7:3, *v*/*v*, respectively, at a 1 mL/min flow rate. The eluate was monitored at 273 nm, and the detection limit was about 0.23 µg/mL under our experimental conditions. Accuracy and relative standard deviations were 98.5% and 0.42%, respectively. The chromatographic peaks of RLZ (using mobile phase A), and RAS, SAR, and SGR (using mobile phase B) were detected at 6.9 min, 6.5 min, 4.9 min, and 4.6 min, respectively.

### 2.4. Distribution Coefficient and Chemical Stability

To measure distribution coefficients of test compounds in 1-octanol/pH 6.8 isotonic phosphate buffer system, 1-octanol and pH 6.8 isotonic phosphate buffer was pre-saturated with each other. Test compounds were dissolved in 10 mL of the 1-octanol phase (RLZ, RAS, AR, GR) or 10 mL of the aqueous phase (SAR and SGR), followed by combining the two phases and shaking the mixtures at 200 rpm on an orbital shaker for 12 h. Distribution of test compounds in the organic phase/aqueous phase was determined by quantifying the concentration of each test compound in the organic or aqueous phase using a UV-Vis spectrophotometer (Shimadzu, Tokyo, Japan) at 263 nm. The following equation was employed to calculate the distribution coefficients (log *D*_6.8_). In the equation, *C*_O_ is the initial concentration of a compound in the 1-octanol phase or aqueous phase; *Cw* is the equilibrium concentration of a compound in the aqueous phase; and *C_O_c* is the equilibrium concentration of a compound in 1-octanol:

$$\log\;D_{6.8}=\log(C_{Oc}/C_{W})=\log\left[(C_{Oc}/(C_{O}-C_{Oc}\right)]$$

To test the chemical stabilities of SAR and SGR, the two compounds (0.1 mM) were incubated for 12 h in pH 1.2 HCl-NaCl buffer and pH 6.8 isotonic phosphate buffer, and change in the drug concentrations was monitored using HPLC analysis.

### 2.5. Cell Permeability Assay

Caco-2 cells (4 × 10^5^ per insert) were seeded in Transwell 6-well inserts with a pore size of 0.4 μm (SPL Inc., Houston, TX, USA). DMEM containing 1% penicillin/streptomycin was added to the basolateral (3.0 mL) and apical (insert) compartments (2.0 mL). The cells were grown until the cell monolayer’s transepithelial electrical resistance value (EMD Millipore, Billerica, MA, USA) reached 2000 Ω.cm^2^. After removing the culture medium from both compartments, RLZ and its derivatives (500 μM) dissolved in DMEM without phenol red (2.0 mL) were placed in the apical compartment of the cell monolayer, while the basolateral compartment was filled with DMEM without phenol red (3.0 mL). At appropriate time intervals, the drug concentration in the basolateral compartment was determined using HPLC.

### 2.6. Animals

Seven-week-old male Sprague-Dawley rats were purchased from Samtako Bio Korea (Gyeonggi-do, Republic of Korea) and housed in an animal care facility at Pusan National University (Busan, Republic of Korea) under controlled temperature, humidity, and dark/light (12 h/12 h) cycle conditions. The animal protocol used in this study was reviewed and approved by the Pusan National University—Institutional Animal Care and Use Committee (PNU–IACUC) for ethical procedures and scientific care (Approval No: PNU-2023-0229, Approval Date: 29 March 2023).

### 2.7. Incubation of Drugs in the Contents of the Small Intestine and the Cecum of Rats

Male Sprague-Dawley rats (250–260 g) were euthanized using CO_2_ gas, collecting the intestinal tract contents separately from the small intestine and cecum. The cecal contents were collected in an atmospheric bag (AtmosBag, Sigma, St. Louis, MO, USA) filled with N_2_ gas. The contents were suspended in pH 6.8 isotonic phosphate buffer (20%, *w*/*v*). To 5 mL of the cecal and small intestinal suspensions, each solution (5 mL) of RAS, SAR, and SGR (2.0 mM) dissolved of pH 6.8 isotonic phosphate buffer was added, and then incubated at 37 °C. Incubation with the cecal contents was conducted in the bag filled with N_2_ gas. A 0.5 mL portion of the mixtures was transferred to a microtube, followed by centrifugation at 10,000× *g* at 4 °C for 10 min. Supernatants (0.3 mL) were subjected to extraction with EA (0.3 mL) and subsequent centrifugation at 10,000× *g* at 4 °C for 7 min. The organic layer (0.2 mL) transferred to a new microtube was evaporated, followed by the addition of mobile phase A (0.2 mL). The concentrations of RLZ were analyzed by injecting 20 μL of the filtrate to HPLC, following filtration through a membrane filter (0.45 μm).

### 2.8. Analysis of Drug Concentration in Blood and Cecum

Male Sprague-Dawley rats fasted for 24 h except for water. The rats were treated orally with RLZ (10 mg/kg), RAS (16.4 mg/kg equivalent to 10 mg/kg of RLZ), or SAR (19.2 mg/kg equivalent to 10 mg/kg of RLZ) in PBS (1.0 mL). At 4, 8, and 12 h after the oral treatment, the plasma (0.3 mL) was obtained from the blood samples (collected via cardiac puncture) centrifuged at 10,000× *g* at 4 °C for 10 min. The plasma (0.25 mL) was extracted with EA (0.7 mL), and the organic layer (0.5 mL) was evaporated and dissolved in mobile phase A or B (0.1 mL). After filtration through a membrane filter (0.45 μm), the filtrate (20 μL) was injected to HPLC. To determine the concentrations of SAR and RAS in the blood, MeOH (0.9 mL) was added to the plasma (0.1 mL) and centrifuged at 10,000× *g* at 4 °C for 10 min. After filtration of the supernatants through a membrane filter (0.45 μm), the filtrate (20 μL) was injected to HPLC.

For determination of drug concentrations in the cecum, pH 6.8 isotonic phosphate buffer was added to cecal contents collected at 4, 8, and 12 h after oral treatment to make a 10% suspension. After centrifugation at 10,000× *g* for 10 min at 4 °C, the supernatant (0.2 mL) was subjected to extraction with EA (0.3 mL). The organic layer (0.2 mL) was evaporated, and the residue was dissolved in the mobile phase A (0.2 mL). After filtration through a membrane filter (0.45 μm), the filtrates (20 μL) were injected to HPLC.

### 2.9. DNBS-Induced Rat Colitis and Evaluation of Anticolitic Effects

Experimental colitis was induced in rats as previously described [15]. The rats were divided into six groups (n = 5 per group) and treated via oral gavage as follows: group 1 (normal group), 1.0 mL of PBS; group 2 (colitis group), 1.0 mL of PBS; group 3 (SSZ-treated colitis group), SSZ (30 mg/kg) in 1.0 mL of PBS; group 4 (RAS-treated colitis group), RAS (16.4 mg/kg equivalent to 10 mg/kg of RLZ) in 1.0 mL of PBS; group 5 (SAR (L)-treated colitis group), SAR (9.6 mg/kg equivalent to 5 mg/kg of RLZ) in 1.0 mL of PBS; and group 6 (SAR (H)-treated colitis group), SAR (19.2 mg/kg equivalent to 10 mg/kg of RLZ) in 1.0 mL of PBS. The rats in each group were treated orally once daily, and the medication began three days after the induction of inflammation, and continued for six days. Twenty-four hours after the last medication, the anticolitic effects of drugs were evaluated assessing macroscopic and microscopic indices. Colonic-damage scores (CDS) were assigned using the modified scoring system [13] shown in Supplementary Table S1. Colonic damage was scored using 4 independent observers blinded to the treatment conditions. Myeloperoxidase (MPO) activity was determined in the inflamed distal colon (4 cm) as previously described [13].

### 2.10. Western Blot Analysis

To prepare samples for Western blot analysis, tissue (distal colon) samples (0.2 g) were minced and homogenized in 2.0 mL of pre-chilled radioimmunoprecipitation assay (RIPA) buffer [15], which was agitated on ice for 0.5 h, and then centrifuged at 10,000× *g* at 4 °C for 10 min. The centrifuged lysates were subjected to protein quantification using the bicinchoninic acid reagent (Thermo Fisher Scientific, Waltham, MA, USA). SDS-PAGE on a 7.5 or 10% gel was used for Western blot analysis of tissue lysates. To detect glycogen synthase (GS), phosphorylated GS (p-GS), cyclooxygenase (COX)-2, and inducible nitric oxide synthase (iNOS), anti-GS (Santa Cruz Biotechnology, Dallas, TX, USA), anti-p-GS (Cell Signaling Technology, Danvers, MA, USA), anti-COX-2 (sc-365374, Santa Cruz Biotechnology), and anti-iNOS (NOS-2) antibody (sc-7271, Santa Cruz Biotechnology) were used. SuperSignal chemiluminescence substrate (Thermo Fisher Scientific, Waltham, MA, USA) was used to visualize bands. α-Tubulin (Santa Cruz Biotechnology) was used as a loading control. The Western blot images were quantified using Image Lab software (version 5.2 build 14; Bio-Rad, Hercules, CA, USA). The mean of the quantified values was presented with the Western blot images in the figures (n = 3 for cell experiments, n = 5 for animal experiments).

### 2.11. ELISA for CINC-3 and IL-10

ELISA kits were used to determine the level of CINC-3 and IL-10 in the inflamed distal colon as described previously [13]. The ELISA was performed according to the manufacturer’s instructions.

### 2.12. Data Analysis

The results are expressed as the mean ± standard deviation (SD). One-way analysis of variance (ANOVA) followed by Tukey’s HSD test or the Mann–Whitney U test (for CDS) was used to test the differences between the groups. Differences were considered statistically significant at *p* < 0.05. 

## 3. Results

### 3.1. Synthesis of SAR and SGR

The synthetic scheme of SAR and SGR and the structure of RAS are shown in Figure 1A,B. RLZ was coupled with the acidic amino acids Asp and Glu, followed by N-succinylation of the amino groups of the amino acids, yielding SAR and SGR. Their formation was verified using IR spectroscopy, 1H-NMR spectroscopy, and mass spectrometry. In the IR spectra (Supplementary Figure S1A), the carbonyl stretching bands of the amide and carboxylic acid resulting from the N-succinylation of the amino acids were observed at 1557 cm^−1^ and 1657 cm^−1^ (for SAR), and 1584 cm^−1^ and 1689 cm^−1^ (for SGR). Other carbonyl bands of the carboxylic acid of the amino acids and the amide bond formed between the aromatic amine of RLZ and the carboxylic acid of the amino acids were observed at 1699 cm^−1^ and 1611 cm^−1^ (for SAR), and 1689 cm^−1^ and 1616 cm^−1^ (for SGR). The two carbonyl bands derived from the carboxylic acids in SGR overlapped in the IR spectrum. In the 1H-NMR spectra of SAR and SGR (Supplementary Figure S1B), aromatic proton signals originating from RLZ were detected at 7.4–8.2 ppm. In addition, aliphatic proton signals of succinylated amino acids were observed between 1.8 and 4.8 ppm. Mass spectrometry revealed molecular peaks corresponding to the molecular weights of SAR and SGR (Supplementary Figure S1C).

### 3.2. SAR but Not SGR Is Colon-Specific

**Figure 2:**
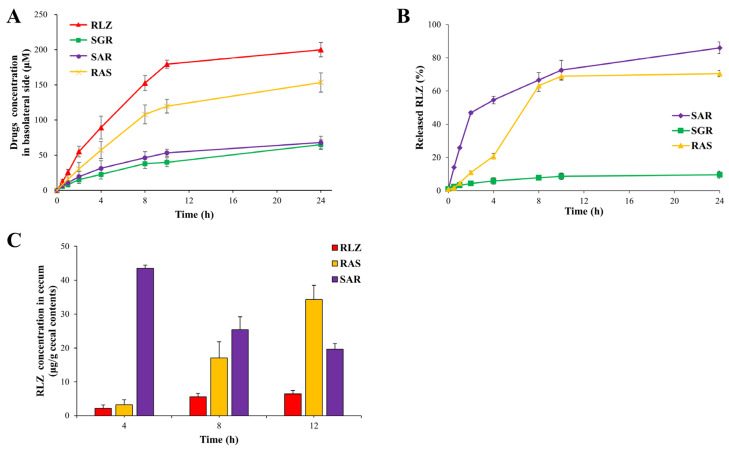
SAR is colon specific. (**A**) RLZ, RAS, SAR, and SGR (500 µM, 2 mL) dissolved in the medium were added to the apical compartment of the Caco-2 cell monolayer and incubated for 24 h. At appropriate time intervals, the concentrations of each drug were determined in the basolateral compartment filled with medium (3 mL) using HPLC. (**B**) RAS, SAR, and SGR (1 mM) were incubated with suspension of the cecal contents (10.0%). The supernatants obtained using centrifugation were extracted with EA, and the organic layer in a new mircotube was evaporated, and then dissolved in mobile phase A. After filtration, the filtrates (20 μL) were injected to HPLC to determine the concentration of RLZ. (**C**) Male Sprague-Dawley rats (250–260 g) were starved for 24 h except for water. Rats were treated orally with RLZ (10 mg/kg), RAS (16.4 mg/kg, equivalent to 10 mg/kg of RLZ), or SAR (19.2 mg/kg, equivalent to 10 mg/kg of RLZ) suspended in PBS (pH 7.4). The concentration of RLZ in the cecum was determined using HPLC at 4, 8, and 12 h after the treatment. The data are represented as the mean ± SD (n = 5).

To examine the colon specificity of SAR and SGR, their chemical and biochemical stabilities during transit in the upper intestine were tested and incubated in buffer solutions at pH 1.2 and 6.8, representing gastric and intestinal pH values, respectively. In addition, incubation of the small intestinal contents was performed. No significant change in their concentrations was observed for 12 h. Consistent with a previous study [14], AR and GR, produced by the simple conjugation of RLZ with acidic amino acids, released a substantial amount of RLZ in the small intestinal contents (Supplementary Figure S2). Distribution coefficients (log *D*_6.8_), which predict systemic absorption via passive transport, were measured using a 1-octanol/isotonic phosphate buffer (pH 6.8) system. The log *D*_6.8_ of RLZ (3.5) was lowered to 0.8 (for AR) and 1.0 (for GR) in conjugation with acidic amino acids and further lowered to −2.1 (SAR) and −2.0 (SGR) with N-succinylation. The results of distribution coefficients indicated that SAR and SGR were more hydrophilic than RAS with a distribution coefficient of 2.1 [13]. To further test cell permeability, SAR, SGR, and RAS were subjected to cell-permeability tests using Caco-2 monolayers. As shown in Figure 2A, N-succinylated conjugates possessing two carboxylic acid groups were more effective in retarding the permeation of RLZ through the cell monolayer than RAS with one carboxylic acid group. These results indicate that the passive transport of N-succinylated conjugates across the intestinal epithelial layer is less efficient than that of RAS, suggesting that the colonic delivery efficiency of N-succinylated conjugates is greater than that of RAS. Next, we examined whether the N-succinylated conjugates delivered to the large intestine were converted to RLZ. SAR and SGR were incubated with the cecal contents, and RLZ concentrations were determined at appropriate time intervals. The cecal contents were incubated in a nitrogen glove bag to maintain anaerobic conditions. For comparison with RAS, the same experiment was conducted using RAS. As shown in Figure 2B, SAR was readily converted to RLZ in a time-dependent manner, whereas SGR was resistant to cecal conversion of RLZ. Compared with RAS (11% at 2 h, 63% at 8 h, and 70% at 24), SAR showed a higher conversion profile (47% at 2 h, 67% at 8 h, and 86% at 24). These in vitro results suggest that SAR, but not SGR, acts as a colon-targeted prodrug and elicits improved colon-targeting properties compared to RAS. To verify this in vivo, SAR was administered orally to rats, and RLZ concentrations were determined in the cecum at 4, 8, and 12 h after oral administration. For comparison, the same experiment was conducted using RAS and RLZ. As shown in Figure 2C, oral administration of SAR and RAS, enabling colon-targeted delivery of RLZ, resulted in accumulating a greater amount of RLZ in the cecum than that of RLZ. In addition, SAR showed greater cecal accumulation of RLZ than RAS at 4 and 8 h, and the maximal concentration of RLZ obtained after oral SAR was higher than that obtained after oral RAS. Additionally, the overall amount of RLZ detected in the cecum was greater with oral SAR than with oral RAS. Consistent with the in vitro results, these results indicate that SAR improves the colon-targeting properties of RAS for the colonic delivery of RLZ.

### 3.3. SAR Is More Effective against DNBS-Induced Rat Colitis than RAS

**Figure 3:**
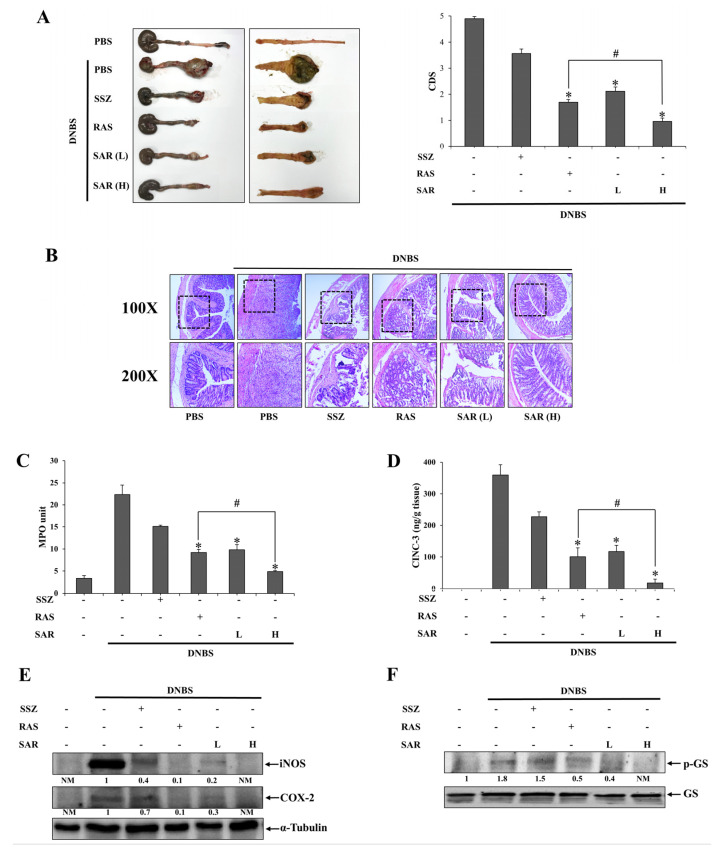

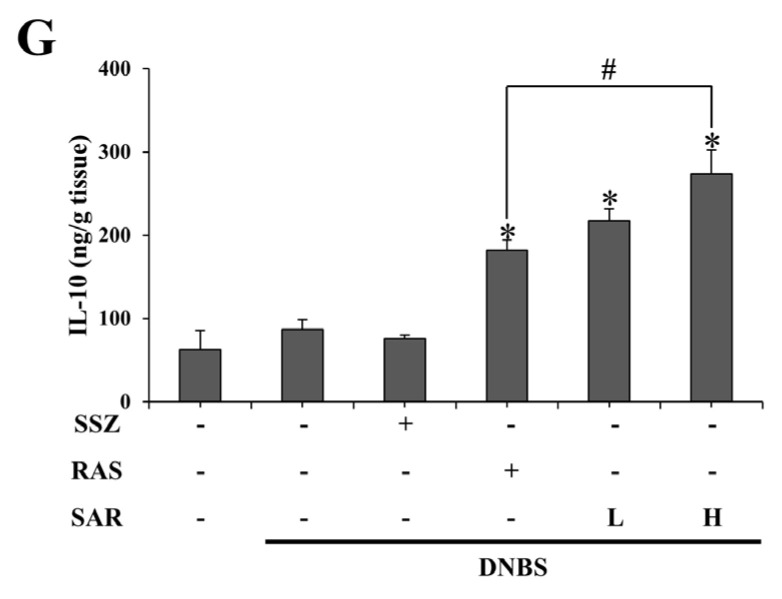
SAR is more effective in reducing systemic absorption of RLZ than RAS. Rats were treated orally with SSZ (30 mg/kg), RAS (16.4 mg/kg, equivalent to 10 mg/kg of RLZ), SAR (L) (9.6 mg/kg, equivalent to 5 mg/kg), and SAR (H) (19.2 mg/kg, equivalent to 10 mg/kg) dissolved in 1.0 mL of PBS (pH 7.4) once per day, and continued for 6 days. The medications began 3 days after induction of colitis using DNBS. Anticolitic effects of the drugs were evaluated 24 h after the last dose. (**A**) Left panel: Images of the serosal and luminal sides of the distal colons of rats. Right panel: Colonic-damage score (CDS) was assigned for each treatment. (**B**) The colonic tissue sections of rats were subjected to H & E staining. Upper panel: Representative images of 100× magnification. Lower panel: Representative images of 200× magnification for the dotted boxes in the upper panel. (**C**) Myeloperoxidase (MPO) activity, (**D**) CINC−3 level, and (**E**) iNOS and COX−2 level in the inflamed distal colons (4.0 cm) were assessed. For Western blot analysis, α−Tubulin was used as a loading control to normalize iNOS and COX−2 levels. NM: not measurable. (**F**) The levels of p−GS and GS in the inflamed distal colon were monitored using Western blot analysis. NM: not measurable. (**G**) The levels of IL10 in the inflamed distal colon were determined using an ELISA kit. The data are represented as mean ± SD (n = 5). * *p* < 0.05, vs. DNBS control ^#^ *p* < 0.05.

Our data strongly suggest that the SAR supplies a greater concentration of RLZ at the inflamed site (large intestine) than the RAS. We examined whether the improved colonic delivery efficiency was linked to greater anticolitic effects. SAR (equivalent to 5 (L) and 10 (H) mg/kg RLZ) was administered orally to DNBS-induced colitic rats. The same experiment was conducted using RAS (equivalent to 10 mg/kg RLZ) and SSZ (30 mg/kg RLZ). Colonic damage and inflammation were evaluated using macroscopic and microscopic indices, including CDS, H & E staining; MPO activity; and inflammatory mediators CINC-3, COX-2, and iNOS levels in the inflamed distal colon. As shown in Figure 3A–E, DNBS induced severe colonic damage and inflammation with pathological lesions, such as ulcers with hemorrhagic scabs, erosion of the mucosal layer, edema, stricture, adhesion with neighboring organs, and shortening of the colon. SAR mitigated the pathological state of the inflamed colon at both doses (L and H), as shown in the tissue images of CDS (Figure 3A), and H&E staining of inflamed distal colons (Figure 3B). In parallel, SAR lowered MPO activity (Figure 3C) and the levels of inflammatory mediators CINC-3 (Figure 3D), COX-2, and iNOS (Figure 3E) in a dose-dependent manner. SAR (even at a lower dose) was more effective than SSZ against colonic damage and inflammation. Moreover, at an equimolar dose (equivalent to 10 mg/kg RLZ), SAR was significantly superior to RAS in improving all anticolitic indices, indicating that improved colon-targeted properties led to greater potency against colitis. Moreover, since anticolitic activity of colon-targeted RLZ is relevant in RLZ inhibition of GSK3β [13], levels of phosphorylated glycogen synthase (p-GS) were determined in the inflamed colons. GS is a substrate of GSK3β, and at the same time, the anti-inflammatory cytokine IL-10 levels induced by the inhibition of GSK3β [14,16] were also determined in the inflamed colon. As shown in Figure 3F,G, RAS, and SAR substantially diminished the level of p-GS, and SAR was similarly effective to RAS at an equimolar dose. In parallel, SAR and RAS further augmented the level of the anti-inflammatory cytokine IL-10, which was elevated by the induction of inflammation. SAR showed a greater ability to augment the level of IL-10 than RAS at an equimolar dose.

### 3.4. SAR Is More Effective in Reducing Systemic Absorptionof RLZ than RAS

**Figure 4:**
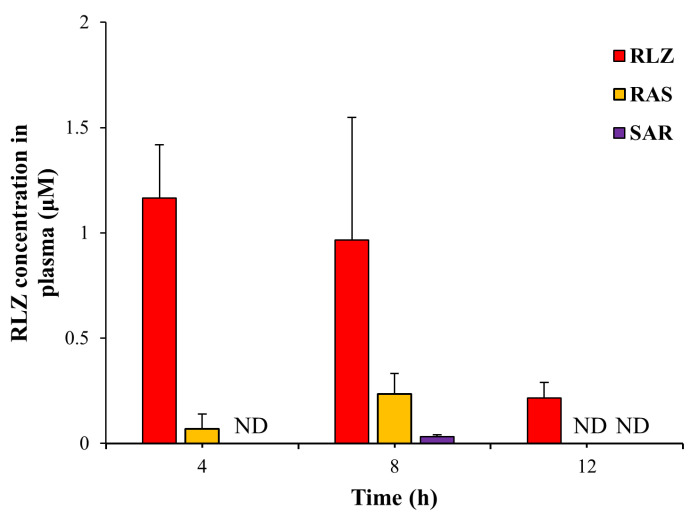
SAR has a greater ability to restrict systemic absorption of RLZ than RAS.

Limiting the systemic absorption of a parent drug, likely leading to a reduced risk of systemic side effects, is considered an advantage of CTDD, which confers a toxicological benefit (safety) to a colon-targeted prodrug. In our previous study [13], oral RAS lowered the peak concentration of RLZ in the blood to approximately one-fifth of that obtained after oral RLZ. We examined whether SAR, which is more hydrophilic and less permeable to the epithelial cell layer than RAS, could further reduce the maximum blood concentration of RLZ. The concentration of RLZ in the blood was determined at 4, 8, and 12 h after oral SAR. The same experiment was performed using equimolar doses of RAS and RLZ. As shown in Figure 4, consistent with a previous study [13], the peak concentration of RLZ was observed 4 h after oral administration of RLZ, and oral RAS lowered the peak concentration of RLZ to approximately one-fifth. SAR further reduced the systemic absorption of RLZ to the extent that it was barely detectable in the blood. These results indicate that upon repositioning RLZ to an anticolitic drug, SAR may be safer than RAS in terms of the systemic side effects of RLZ.

Male Sprague-Dawley rats (250–260 g) were starved for 24 h except for water. Rats were treated orally with RLZ (10 mg/kg), RAS (16.4 mg/kg, equivalent to 10 mg/kg of RLZ), or SAR (19.2 mg/kg equivalent to 10 mg/kg of RLZ) in PBS (pH 7.4). The concentration of RLZ in the blood was determined using HPLC at 4, 8, and 12 h after the oral treatments. The data are represented as the mean ± SD (n = 5). ND in the figure indicates “not detectable” in our analysis condition.

## 4. Discussion

RLZ is an FDA-approved anti-amyotrophic lateral sclerosis drug. Since its potential molecular mechanisms such as the inhibition of NMDA receptors and activation of the Wnt/β-catenin pathway are utilizable as therapeutic targets for the development of anti-IBD drugs [16,17,18,19], RLZ was subjected to drug repositioning to an anticolitic drug [13]. For the successful repositioning of RLZ as an anticolitic drug, a pharmaceutical strategy, CTDD, was employed. Indeed, RAS, designed and synthesized as a colon-targeted prodrug of RLZ, enhances the anticolitic activity and reduces the risk of systemic side effects, thus facilitating the repositioning of RLZ [13]. However, considering that RAS has a relatively high distribution coefficient (not sufficiently hydrophilic to prevent systemic absorption of RAS) and is detectable in the blood after oral administration [13], it is reasonable to hypothesize that a colon-targeted prodrug of RLZ with greater hydrophilicity would improve the colon specificity of RAS, likely leading to improved safety and therapeutic potency.

Coupling the carboxylic acid group of the N-succinylated forms of acidic amino acids with a sulphonamide group is a feasible way to design a colon-targeted prodrug of celecoxib [14]. This chemical modification made the conjugate highly hydrophilic because of the presence of two free carboxylic groups. At the same time, the chemical link between celecoxib and amino acids, a carbonyl sulfonamide linker, is susceptible to metabolism by the microbial enzyme(s) in the large intestine, leading to its conversion back to celecoxib. Therefore, this design strategy was employed to improve colon specificity of the RAS. Our data show that the design strategy was effective for RLZ with an amine group instead of a sulfonamide group. However, unlike celecoxib conjugated with N-succinylated acidic amino acids, where the celecoxib conjugates are metabolized to celecoxib in the cecal contents regardless of the N-succinylated carriers [14], RLZ conjugated with N-succinylated acidic amino acids showed different metabolic susceptibilities in the cecal contents. SAR, produced with the conjugation of RLZ with N-succinylated Asp, was easily converted to RLZ, while SGR, produced with the conjugation of RLZ with N-succinylated Glu, was resistant to conversion to RLZ in the cecal contents, suggesting that microbial amidases in the cecal contents distinguish the two conjugates as substrates. Nonetheless, consistent with the colon-targeted prodrug of celecoxib conjugated with N-succinylated Asp, cleavage of SAR (to produce RLZ) occurred via a one-step rather than a two-step process. Cleavage of the amide bond between RLZ and N-succinylated Asp is the predominant metabolic pathway that generates RLZ in cecal contents. In support of this argument, although AR was also metabolized to RLZ in the cecal contents, its conversion percentage to RLZ was lower than that of SAR (Supplementary Figure S3), and no AR was detected during the incubation of SAR with the cecal contents. Therefore, colonic activation of SAR to RLZ occurs largely with a one-step cleavage of succinylated Asp from SAR rather than by stepwise cleavage of the two amide bonds in SAR, thus forming AR as an intermediate.

As expected from the design of the prodrugs, SAR with two carboxylic groups was more hydrophilic than RAS with one carboxylic group, as assessed using the distribution coefficients. In parallel, in cell-permeability tests using Caco-2 cell monolayers, SAR was less efficient than RAS in permeating the cell monolayer. Consistent with these results, oral SAR resulted in greater cecal accumulation of RLZ than oral RAS, suggesting that SAR is more efficient in delivering RLZ to the large intestine than RAS. This was confirmed by comparing the blood concentrations of RAS and SAR. After oral administration of SAR and RAS at an equimolar dose, SAR was not detectable in the blood, whereas RAS was detected (Supplementary Figure S4).

Temporal changes in the cecal accumulation of RLZ after oral SAR were different from those after RAS, as shown in Figure 2C. Oral SAR accumulated a greater amount of RLZ at earlier time points (4 and 8 h), whereas a greater amount of RLZ was observed at the final time point (12 h) with oral RAS. This different accumulation profile of RLZ is likely due to the difference in the hydrophilicity of SAR and RAS, although we cannot rule out differences in cecal conversion rates. Unlike the experiment using cecal contents, in which RAS and SAR dissolved in a buffer solution were incubated, RAS and SAR suspended in a buffer solution, where part of the prodrugs was dissolved, were administered. In this case, the dissolution of prodrugs in the large intestine is required for conversion to RLZ, and the conversion of RAS, which is less hydrophilic than SAR, may occur more slowly than that of SAR. In addition, SAR, whose larger part is likely to be dissolved in the upper intestine than RAS, may reach the cecum earlier than RAS because the gastric transit time of the solution state is shorter than the non-solution state [20].

Our data showed that the colonic delivery efficiency was linked to the anticolitic activity of SAR and RAS. SAR is more effective than RAS in mitigating colonic damage and inflammation. SAR healed colonic damage induced by DNBS, as assessed by CDS and H&E staining, in addition to improving colon shortening. In parallel, SAR lowered MPO activity and the levels of inflammatory mediators, such as CINC-3, iNOS, and COX-2, in the inflamed colon. All the anticolitic effects of SAR were significantly superior to those of RAS. In addition to the higher anticolitic potency of SAR, it is very likely that SAR elicits improved safety compared to RAS. This argument is supported by the data demonstrating that SAR exhibited a greater ability to limit the systemic absorption of RLZ than RAS, as shown in the comparison of blood concentrations of RLZ after oral administration of SAR and RAS. Generally, the blood concentration of a parent drug indicates the systemic side effects elicited by a colon-targeted prodrug [13]. Given that a larger amount of RLZ accumulates in the cecum with oral SAR, data on blood concentrations of RLZ suggest that hydrophobic RLZ is barely absorbed in the large intestine, where the water content is relatively low compared to other parts of the GI tract [21]. Therefore, most of the RLZ in the blood after oral RAS administration is unlikely to originate from the large intestine. Since RAS was detected in the blood as mentioned above, and compounds with an azo bond can be metabolized (reduced) in the liver [22], RLZ in the blood may result from the hepatic metabolism of RAS that is absorbed systemically.

## 5. Conclusions

SAR, synthesized by coupling RLZ with N-succinylated Asp via an amide bond, acts as a colon-targeted prodrug of RLZ. SAR, synthesized by coupling RLZ with N-succinylated Asp via an amide bond, acts as a colon-targeted prodrug of RLZ. Compared with oral RAS, oral SAR accumulated at approximately 1.8-fold the amount of RLZ in the large intestine (between 4 h and 12 h after treatment) and reduced the maximal plasma concentration of RLZ up to approximately one-seventh. Furthermore, SAR was more effective than RAS in improving all the anticolitic indices with statistical significance. These results suggest that SAR exhibits superior anticolitic potency and safety over RAS, an azo-type colon-targeted prodrug of RLZ.

## Data Availability

The data presented in this study are available in the article or Supplementary Materials.

## Supplementary Materials

The following supporting information can be downloaded at https://www.mdpi.com/article/10.3390/pharmaceutics15112638/s1, Table S1: Modified scoring system. Figure S1: Instrumental characterization of SAR and SGR. (A) FT-IR spectra of SAR and SGR, (B) 1H-NMR spectra of SAR and SGR, (C) Mass spectra of SAR and SGR. Figure S2: Stability of SAR, SGR, AR and GR in the small intestinal contents. SAR, SGR, AR and GR were incubated with the small intestinal contents suspended in PBS (pH 6.8, 10.0%). The concentrations of RLZ were analyzed by HPLC at a predetermined time interval. Figure S3: Conversion of AR and SAR to RLZ in the cecal contents. SAR and AR (1 mM) were incubated with the cecal contents suspended in PBS (pH 6.8, 10.0%). The concentration of RLZ was analyzed by HPLC at a pre-determined time interval. Figure S4: Concentrations of the conjugates in the blood after oral administration of RAS and SAR to rats. Male Sprague-Dawley rats (250–260 g) were starved for 24 h except for water. Rats were treated orally with RAS (16.4 mg/kg, equivalent to 10 mg/kg of RLZ) and SAR (19.2 mg/kg, equivalent to 10 mg/kg of RLZ) in PBS (pH 7.4). The concentrations of RAS and SAR in the blood were determined using HPLC at 4, 8, and 12 h after the oral treatments.
