# Supplementary material for: N-Succinylaspartic-Acid-Conjugated Riluzole Is a Safe and Potent Colon-Targeted Prodrug of Riluzole against DNBS-Induced Rat Colitis
Source: Pharmaceutics. 2023 Nov 16;15(11):2638. doi: 10.3390/pharmaceutics15112638 (PMC10675528; doi:10.3390/pharmaceutics15112638)
Supplement: Supplementary file 1 [file pharmaceutics-15-02638-s001.zip › pharmaceutics-2674033-supplementary.pdf]

Supplementary Table S1. Modified scoring system

Score	Feature
0	normal appearance
1	localized hyperemia but no ulcer
2	linear ulcers without significant inflammation
3	2–4 cm site of inflammation and ulceration
4	serosal adhesion to other organs, 2–4 cm site of inflammation and ulceration
5	stricture, serosal adhesion involving several bowel loops, <4 cm site of inflammation and ulceration

Supplementary Figure S1. Instrumental characterization of SAR and SGR

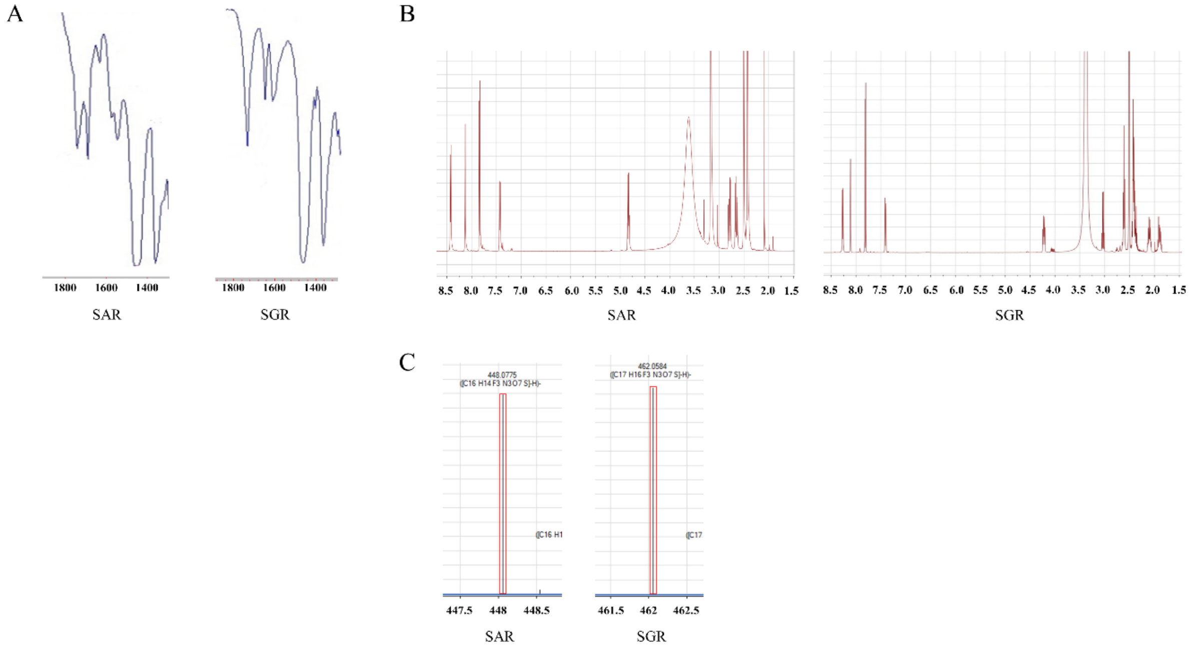

(A) FT-IR spectra of SAR and SGR

(B) ^{1}H-NMR spectra of SAR and SGR

(C) Mass spectra of SAR and SGR

Supplementary Figure S2. Stability of SAR, SGR, AR and GR in the small intestinal contents

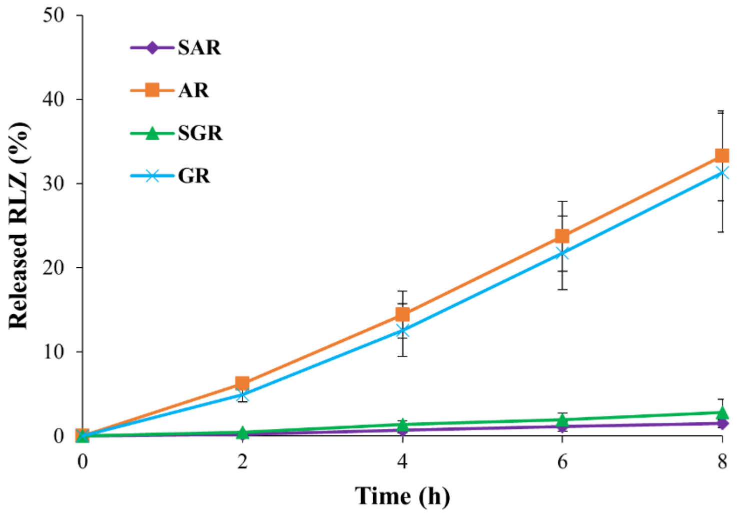

SAR, SGR, AR and GR were incubated with the small intestinal contents suspended in PBS (pH 6.8, 10.0%). The concentrations of the RLZ was analyzed by HPLC at a predetermined time interval.

Supplementary Figure S3. Conversion of AR and SAR to RLZ in the cecal contents

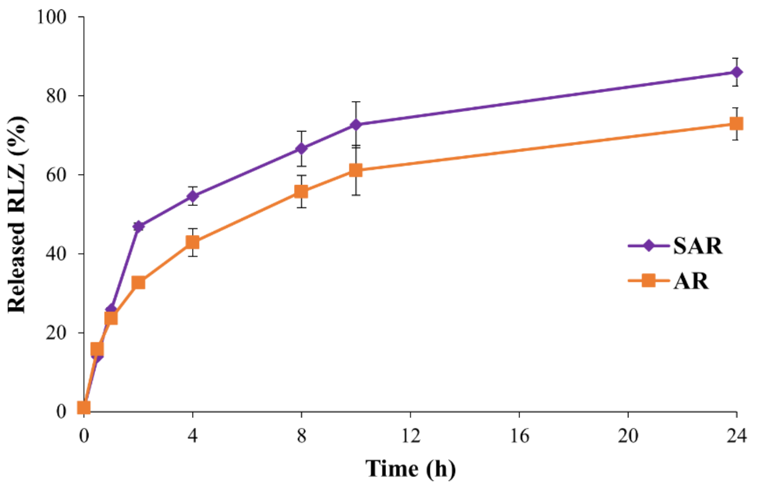

SAR and AR (1 mM) were incubated with the cecal contents suspended in PBS (pH 6.8, 10.0%). The concentration of RLZ was analyzed by HPLC at a pre-determined time interval.

Supplementary Figure S4. Concentrations of the conjugates in the blood after oral administration of RAS and SAR to rats

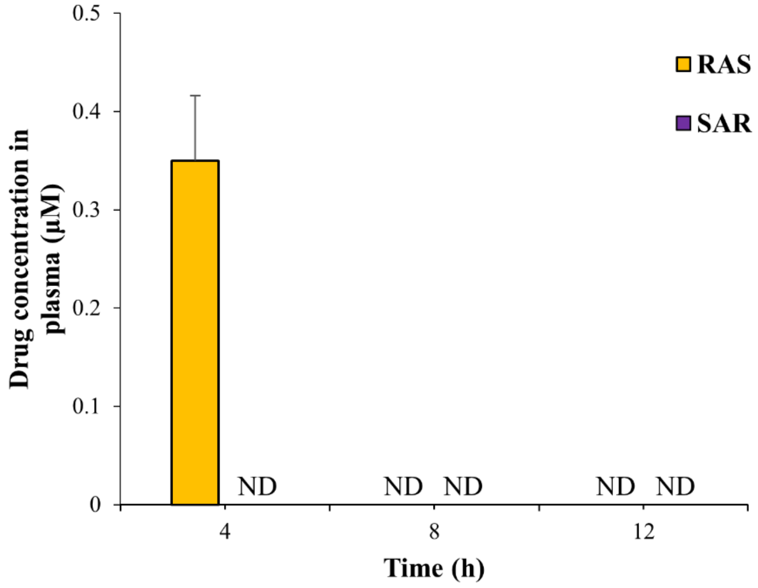

Male Sprague-Dawley rats (250–260 g) were starved for 24 h except for water. RAS (16.4 mg/kg, equivalent to 10 mg/kg of RLZ) and SAR (19.2 mg/kg, equivalent to 10 mg/kg of RLZ) suspended in PBS (pH 7.4) was administered to rats by oral gavage. The rats were sacrificed 4, 8, and 12 h after the oral gavage, and the concentration of RAS and SAR in the blood were analyzed using HPLC.
